# Surgical management of presternal recurrence in hyperparathyroidism related to chronic kidney disease

**DOI:** 10.1016/j.bjorl.2026.101925

**Published:** 2026-10-14

**Authors:** Murilo Catafesta das Neves, Giovanna Luiza Caxeiro, Davi Knoll Ribeiro, Marcello Rosano, Lillian Andrade da Rocha, Aluizio Barbosa Carvalho, Rodrigo Oliveira Santos

**Affiliations:** a Universidade Federal de São Paulo (UNIFESP), Department of Otorhinolaryngology and Head and Neck Surgery, São Paulo, SP, Brazil; b Universidade Federal de São Paulo (UNIFESP), Department of Mineral and Bone Disorders, Discipline of Nephrology, São Paulo, SP, Brazil

**Keywords:** Hyperparathyroidism, Secondary, Parathyroidectomy, Chronic kidney disease-mineral and bone disorder, Autografts, Reoperation

## Abstract

**Objective:**

To evaluate the surgical management and outcomes of recurrence localized in presternal autografts after total parathyroidectomy with autograft for hyperparathyroidism related to chronic kidney disease.

**Methods:**

A retrospective cohort study was conducted including patients submitted to total parathyroidectomy with presternal autograft between 2000 and 2024 who developed biochemical recurrence according to Brazilian Society of Nephrology criteria and required reoperation. Only cases occurring more than six months after an initially effective surgery were included. The primary outcome was sustained biochemical resolution.

**Results:**

Twenty-four patients required reoperation due to recurrence in the presternal autograft. All were on hemodialysis at initial surgery. Mean CKD duration was 96-months and mean preoperative PTH level was 1445.4 pg/mL. Thirty-one reoperations were performed; most patients (87.5%) required only one procedure. The mean interval between initial surgery and first graft reoperation was 62.6-months. No invasive growth beyond anatomical limits was observed. Mean intraoperative PTH reduction was 80.8%. Considerable variability was observed between intraoperative PTH reduction and subsequent clinical outcomes. Total graft removal was feasible when indicated.

**Conclusion:**

Reoperation for recurrence in presternal autografts was feasible in this series and was associated with satisfactory biochemical control, even in patients requiring multiple procedures. The anatomical characteristics of the presternal region allowed controlled resections without evidence of invasive growth beyond the graft bed.

**Level of Evidence:**

3.

## Highlights

• Presternal autograft recurrence can be safely managed surgically.
• No invasive growth beyond anatomical limits was observed.
• Most patients required only one reoperation.
• Presternal location provides clear anatomical boundaries.
• Total graft removal was feasible when indicated.

## Introduction

Hyperparathyroidism related to Chronic Kidney Disease (HPT-CKD) is a prevalent complication in patients undergoing hemodialysis and follows a progressive course.^1^ With the development of mineral and bone disorders, vascular and interstitial calcifications, anemia, immunodeficiency, bone pain, and fractures may occur.^2^^,^^3^ Treatment aims to control metabolic disturbances, including the management of Parathyroid Hormone (PTH) levels.^1^

Although the ideal PTH levels for each stage of CKD remain uncertain,^2^ persistently elevated PTH values are associated with a significant increase in morbidity and mortality, as well as refractoriness to medical treatment.^1^^,^^4^^,^^5^

Parathyroidectomy (PTX) is highly effective in reducing PTH levels and correcting metabolic disturbances associated with HPT-CKD, promoting improvement in quality of life and substantial reduction in mortality.^4^, ^5^, ^6^, ^7^

Total Parathyroidectomy with immediate heterotopic Autotransplantation (PTX-AT) is one of the surgical options. It consists of removal of all parathyroid tissue from the cervical region and implantation of an autograft in a muscular bed, most commonly the brachioradialis muscle.^8^

However, persistence of CKD and/or selection of nodular hyperplastic tissue may favor recurrence of HPT-CKD at the Autotransplantation (AT) site.^9^^,^^10^ Recurrence in the upper limb has been widely discussed in the literature and occurs in approximately 8% of patients.^10^ Several authors have reported difficulties in its surgical management, sometimes requiring extensive resections^10^, ^11^, ^12^, ^13^, ^14^ due to uncontrolled and infiltrative growth of the grafted tissue.

For this reason, since the early 2000s, our group has systematically performed AT in the presternal region, adjacent to the pectoralis major muscle, in a single muscular bed^15^ to avoid such complications.

The present study aims to analyze cases of HPT-CKD recurrence in the presternal region and to provide guidance regarding its surgical management, evaluating feasibility and safety.

## Methods

This retrospective cohort study included patients submitted to PTX with presternal AT between 2000 and 2024 who developed recurrence during follow-up. All patients were operated on by the Head and Neck Surgery team at two referral centers.

Recurrence of HPT-CKD was defined based on biochemical criteria established by the Brazilian Society of Nephrology (SBN) guidelines.^5^ Indication for reoperation was established in cases of refractory recurrence despite medical management, following multidisciplinary evaluation.

Only cases occurring more than six months after an initially effective PTX-AT were considered recurrence. Initial surgery was defined as effective when at least four parathyroid glands were removed, with intraoperative PTH (PTHio) reduction greater than 70% from baseline and/or normalization or sustained reduction of serum levels in the immediate postoperative period.^16^

The location of recurrence was determined by physical examination (presence of a palpable mass) and/or compatible imaging studies, including sestamibi scintigraphy showing increased uptake and ultrasonography demonstrating a nodular lesion in the presternal region, characterizing recurrence localized in the AT. Patients with recurrence localized in the cervical region were not included in the present analysis.

Demographic and laboratory data were collected for descriptive analysis of the sample.

For reoperation, partial AT resection was performed. The graft bed was opened with dissection of tissue planes and separation of the autograft from the pectoralis major muscle. The medial portion of the graft adjacent to the sternum was not dissected to preserve its vascularization and viability. The ribs above and below the graft limited the dissection. The lesion was sectioned using a scalpel blade, preserving the medial portion equivalent in volume to two normal parathyroid glands. Intraoperative PTH levels were collected for further analysis.

All patients were followed for at least six months to assess procedural effectiveness, defined as sustained biochemical resolution of recurrence without the need for further surgical intervention. All patients signed informed consent and agreed to participate in the study. Approval was obtained from the Institutional Research Ethics Committee (UNIFESP) and registered on UNIFESP/EPM (691.538).

## Results

During follow-up, 24 patients required surgical reintervention for recurrent HPT-CKD localized in the presternal AT.

At the time of the initial PTX-AT, all 24 patients were receiving hemodialysis as renal replacement therapy. The mean duration of CKD was 96-months, and the mean PTH level was 1445.4 ± 458.2 pg/mL. Patient demographic and clinical characteristics at the time of the initial PTX-AT are summarized in Table 1.

**Table 1:** Demographic, clinical, and operative characteristics at the time of the initial parathyroidectomy with Presternal Autotransplantation (PTX-AT).

Variable	Value
Number of patients	24
Age, years	42.0 ± 9.0 (25–57)
Female sex, n (%)	16 (66.7)
Male sex, n (%)	8 (33.3)
**Race/Ethnicity, n (%)**	
Black	10 (41.7)
White	14 (58.3)
**Cause of chronic kidney disease, n (%)**	
Unknown	6 (25.0)
Hypertensive nephropathy	5 (20.8)
Chronic glomerulonephritis	5 (20.8)
Other causes	5 (20.8)
Diabetic nephropathy	3 (12.5)
Patients undergoing conservative treatment before dialysis, n (%)	5 (20.8)
Duration of conservative treatment, months^a^	39.0 ± 46.7 (4–132)
Duration of hemodialysis, months	96.0 ± 56.2 (6–264)
PTH, pg/mL	1445.4 ± 458.2
Ionized calcium, mmoL/L	1.32 ± 0.12
Phosphorus, mg/dL	6.40 ± 1.78
Alkaline phosphatase, U/L	379.8 ± 438.8

Data are presented as mean ± standard deviation (range) or n (%).

CKD, Chronic Kidney Disease; PTH, Parathyroid Hormone. Reference laboratory values: PTH 10–65 pg/mL, ionized calcium 1.12–1.32 mmoL/L, phosphorus 2.5–4.5 mg/dL, and alkaline phosphatase 40–129 U/L.

a Calculated among the five patients who underwent conservative treatment before initiation of dialysis.

A total of 31 AT reoperations were performed. Most patients (21/24, 87.5%) required only one reintervention. However, two patients (8.3%) underwent three reinterventions, and one patient (4.2%) required four reinterventions due to recurrent graft hyperfunction.

No invasive growth beyond the anatomical boundaries of the graft bed was observed. In all cases, graft regrowth remained confined within the presternal compartment, limited by the sternum and adjacent ribs, with no evidence of pleural or other intrathoracic involvement.

The mean time from initial PTX-AT to the first autograft reoperation was 62.6 ± 37.5 months (range 15–168 months).

At the time of the first AT reoperation, the mean intraoperative PTH reduction was 80.8% ± 11.8% (range 49.2–93.1). Considerable variability was observed between intraoperative PTH reduction and subsequent clinical course. The lowest reduction (49.2%) occurred in a patient who required only one reintervention, whereas the highest reduction (93.1%) was observed in a patient who subsequently required two additional AT reoperations.

Three patients required multiple AT reinterventions. The intervals between successive procedures are shown in Table 2. While the first recurrence occurred 24-, 120-, and 206-months after the initial PTX-AT, subsequent recurrences developed over considerably shorter intervals, with a mean time of 11.9-months (range 3.5–41 months) between consecutive reoperations.

**Table 2:** Time intervals from initial PTX-AT to first recurrence and between subsequent autograft reoperations.

Patient	Initial PTX-AT to 1st recurrence (months)	1st to 2nd (months)	2nd to 3rd (months)	3rd to 4th (months)
1	24	7	3.5	‒
2	120	4	9	41
3	206	8	11	‒

Patient 1 underwent complete AT excision during the third reoperation, followed by implantation of previously cryopreserved parathyroid tissue. This patient remained hypoparathyroid throughout follow-up and was still receiving supplementation 86-months after the last procedure.

Patient 2 died due to abdominal carcinomatosis 11-months after the fourth AT reoperation.

Patient 3 underwent complete AT excision during the third reoperation, developed permanent hypoparathyroidism and was lost to follow-up 10-months after his last intervention.

Renal transplantation was performed in five patients. In three cases, transplantation preceded the first autograft reoperation by 12-, 18-, and 36-months, respectively, and all had a functioning renal graft at the time of recurrence treatment. In the remaining two patients, renal transplantation was performed after autograft reoperation.

## Discussion

Surgical reintervention for recurrence localized in the presternal AT was feasible in our series and was associated with satisfactory biochemical control. No cases of invasive growth beyond anatomical boundaries of the graft bed were observed, allowing controlled partial resections and, when necessary, complete AT excision.

The benefits of surgery in controlling PTH levels are well established in the literature. Rapid correction of metabolic disturbances associated with HPT-CKD leads to improvement in quality-of-life indicators and substantial reduction in mortality.^4^, ^5^, ^6^, ^7^

Wells et al., in their sequential studies,^8^^,^^17^, ^18^, ^19^ standardized the concepts of PTX-AT. Heterotopic autograft implantation in the brachioradialis muscle aimed to reduce the occurrence of permanent hypoparathyroidism and, in case of recurrence, to facilitate surgical access while avoiding new cervical exploration. This topography also allowed measurement of PTH in both arms (with and without graft) to determine a gradient and evaluate AT function.^20^

However, several authors reported difficulties in obtaining and interpreting this gradient.^10^^,^^21^, ^22^, ^23^, ^24^ Others proposed arm tourniquet techniques with complex analytical criteria and undesirable adverse effects.^20^ Thus, the PTH gradient method did not prove practical or universally applicable.

Recurrence of HPT-CKD in the AT occurs in approximately 6%–9% of patients.^10^^,^^22^^,^^24^^,^^25^ As with HPT-CKD, recurrence is closely related to time, with longer CKD duration associated with higher incidence, reaching up to 30% in patients more than 10-years after initial PTX.^24^

The present findings expand our previous experience with presternal autotransplantation. In a prospective series of 66-patients, Santos et al.^26^ reported six graft-dependent recurrences and suggested that graft excision could be performed with relative technical simplicity. However, that study was not specifically designed to evaluate the anatomical behavior of recurrent grafts or the outcomes of repeated reoperations. The current study focuses exclusively on patients who developed recurrent presternal graft hyperfunction and provides additional information regarding recurrence patterns, reoperative management, and long-term outcomes.

Only five patients in our series underwent renal transplantation. Recurrences occurred in this subgroup, which does not allow inference of a protective effect of transplantation. However, the small number of cases precludes adequate statistical analysis, and this observation should be interpreted cautiously.

The choice of the autotransplantation site may influence the complexity of future reoperations. Echenique and colleagues^27^^,^^28^ highlighted the technical difficulties associated with recurrent parathyroid grafts implanted in muscular forearm beds, where diffuse tissue infiltration may hinder complete excision and occasionally require repeated procedures. Based on these concerns, they proposed presternal subcutaneous implantation as an alternative site, arguing that adipose tissue more closely resembles the native parathyroid environment and may facilitate graft localization and removal in cases of recurrence.

Although our technique differs from the subcutaneous approach described by Echenique et al., the present findings support the concept that presternal implantation may facilitate the surgical management of recurrent graft hyperfunction. In our series, recurrent graft tissue remained confined within well-defined anatomical boundaries, no invasive growth beyond the graft bed was observed, and complete AT excision was feasible when required.

Similarly to our findings, other authors have reported success in AT reoperation, with most patients requiring only one procedure.^10^ As previously mentioned, there are several reports describing major difficulties in forearm reintervention,^10^, ^11^, ^12^, ^13^, ^14^^,^^25^^,^^27^^,^^28^ including invasive growth and absence of defined anatomical limits, leading to extensive resections, recommendations against reoperation,^12^ suggestion of limb amputation,^14^ and even death due to failure of clinical control.^13^

Some aspects of graft quality should be considered. Nodular tissue resulting from monoclonal expansion may generate a hypersecretory and clinically refractory graft.^10^ Our group has previously demonstrated that our patients present more severe disease and poorer parathyroid tissue quality for selection,^9^ which could contribute to higher recurrence rates.

In addition, to reduce the occurrence of permanent hypoparathyroidism, and similarly to other authors,^10^^,^^29^ our group increased the amount of grafted tissue from 2010 onward, reaching 90 mg per patient.^30^ A larger graft volume may increase the chance of monoclonal expansion and consequently recurrence.

Thus, given the severity profile of our patients, one could expect not only a higher number of recurrences but also increased surgical difficulty. Nevertheless, even in patients requiring multiple reinterventions, recurrent presternal grafts remained anatomically localized and could be surgically managed without the extensive resections described in some forearm series.

As this is a retrospective study, there are inherent methodological limitations, including possible selection bias and heterogeneity in follow-up duration. The small number of transplanted patients limits subgroup analysis and prevents robust conclusions regarding transplantation impact.

Additionally, the limited availability of calcimimetics in Brazil until 2018 may have influenced the severity profile of patients referred for surgical treatment during the early years of the cohort, representing a potential temporal confounding factor.^31^

## Conclusion

Surgical reintervention for recurrence localized in the presternal AT was feasible in this series and was associated with satisfactory biochemical control of HPT-CKD, even in cases requiring multiple procedures. The anatomical characteristics of the presternal region allowed controlled resections without evidence of invasive growth beyond the graft bed. Although the results are encouraging, the retrospective design and limited number of cases highlight the need for further studies to better define the role of this graft location in the management of HPT-CKD recurrence.

## ORCID ID

Giovanna Luiza Caxeiro: 0009-0004-0093-0862

Davi Knoll Ribeiro: 0000-0002-2543-3844

Marcello Rosano: 0000-0002-3026-466X

Lillian Andrade da Rocha: 0000-0003-4161-2823

Aluizio Barbosa Carvalho: 0000-0001-5907-7764

Rodrigo Oliveira Santos: 0000-0003-1172-4621

## Declaration of Generative AI and AI-assisted technologies in the writing process

During the preparation of this work, the authors used AI-assisted language editing tools to improve clarity and grammar. The authors reviewed and edited the content and take full responsibility for the final manuscript.

## Funding

This research did not receive any specific grant from funding agencies in the public, commercial, or not-for-profit sectors.

## Data availability

The authors declare that all data are available in repository.

## Declaration of competing interest

The authors declare no conflicts of interest.

## References

[1] Hernandes F.R., Goldenstein P., Custódio M.R. (2021). Treatment of hyperparathyroidism (SHPT). Braz J Nephrol.

[2] Foundation N.K. (2015). KDOQI Clinical Practice Guideline for Hemodialysis Adequacy: 2015 update. Am J Kidney Dis.

[3] Group KDIGO (KDIGO) CMW (2009). KDIGO clinical practice guideline for the diagnosis, evaluation, prevention, and treatment of Chronic Kidney Disease-Mineral and Bone Disorder (CKD-MBD). Kidney Int Suppl.

[4] Montenegro F.L.M., Brescia M.D.G., Nascimento Júnior C.P. (2016). The deceptive concept of hypoparathyroidism and recurrence after parathyroidectomy in dialysis patients: are we offering a Procrustean bed to some patients?. Rev Col Bras Cir.

[5] Rocha L.A., Neves M.C., Montenegro F.L.M. (2021). Parathyroidectomy in chronic kidney disease. J Bras Nefrol.

[6] Lau W.L., Obi Y., Kalantar-Zadeh K. (2018). Parathyroidectomy in the management of secondary hyperparathyroidism. Clin J Am Soc Nephrol.

[7] Alves Filho W., Brescia M.D.G., Magnabosco F.F. (2025). Quality of life after parathyroidectomy in chronic kidney disease-related hyperparathyroidism: a systematic review and meta-analysis. World J Surg.

[8] Wells S.A., Gunnells J.C., Shelburne J.D., Schneider A.B., Sherwood L.M. (1975). Transplantation of the parathyroid glands in man: clinical indications and results. Surgery.

[9] Ohe M., Santos R., Neves M. (2012). Tissue selection with or without stereomicroscopy in surgical treatment for renal hyperparathyroidism. Langenbecks Arch Surg.

[10] Tominaga Y., Matsuoka S., Uno N. (2010). Removal of autografted parathyroid tissue for recurrent renal hyperparathyroidism in hemodialysis patients. World J Surg.

[11] Klempa I., Frei U., Röttger P., Schneider M., Koch K.M. (1984). Parathyroid autografts - morphology and function: Six years’ experience with parathyroid autotransplantation in uremic patients. World J Surg.

[12] Frei U., Klempa I., Schneider M., Scheuermann E.H., Koch K.M. (1981). Tumour-like growth of parathyroid autografts in uraemic patients. Proc Eur Dial Transplant Assoc.

[13] Mallette L.E., Eisenberg K.L., Schwaitzberg S.D., Suki W.N., Noon G.P. (1983). Total parathyroidectomy and autogenous parathyroid graft placement for treatment of hyperparathyroidism due to chronic renal failure. Am J Surg.

[14] Stehman-Breen C., Muirhead N., Thorning D., Sherrard D. (1996). Secondary hyperparathyroidism complicated by parathyromatosis. Am J Kidney Dis.

[15] Neves M.C., Ohe M.N., Rosano M. (2012). A 10-year experience in intraoperative parathyroid hormone measurements for primary hyperparathyroidism: a prospective study of 91 previous unexplored patients. J Osteoporos.

[16] Ohe M.N., Santos R.O., Kunii I.S. (2013). Intraoperative PTH cutoff definition to predict successful parathyroidectomy in secondary and tertiary hyperparathyroidism. Braz J Otorhinolaryngol.

[17] Wells S.A., Burdick J.F., Ketcham A.S., Christiansen C. (1973). Transplantation of the parathyroid glands in dogs. Biochemical, histological, and radioimmunoassay proof of function. Transplantation.

[18] Wells S.A., Gunnells J.C., Leslie J.B., Schneider A.S., Sherwood L.M., Gutman R.A. (1977). Transplantation of the parathyroid glands in man. Transplant Proc.

[19] Wells S.A., Stirman J.A., Bolman R.M., Gunnells J.C. (1978). Transplantation of the parathyroid glands: clinical and experimental results. Surg Clin North Am.

[20] Casanova D., Sarfati E., De Francisco A., Amado J.A., Arias M., Dubost C. (1991). Secondary hyperparathyroidism: diagnosis of site of recurrence. World J Surg.

[21] Rothmund M., Wagner P.K., Schark C. (1991). Subtotal parathyroidectomy versus total parathyroidectomy and autotransplantation in secondary hyperparathyroidism: a randomized trial. World J Surg.

[22] Schlosser K., Sitter H., Rothmund M., Zielke A. (2004). Assessing the site of recurrence in patients with secondary hyperparathyroidism by a simplified Casanova autograftectomy test. World J Surg.

[23] Knudsen L., Brandi L., Daugaard H., Olgaard K., Lockwood K. (1996). Five to 10 years follow-up after total parathyroidectomy and autotransplantation of parathyroid tissue: evaluation of parathyroid function by use of ischaemic blockade manoeuvre. Scand J Clin Lab Invest.

[24] Tominaga Y., Uchida K., Haba T. (2001). More than 1,000 cases of total parathyroidectomy with forearm autograft for renal hyperparathyroidism. Am J Kidney Dis.

[25] Rothmund M., Wagner P.K. (1988). Reoperations for persistent and recurrent secondary hyperparathyroidism. Ann Surg.

[26] Santos R.O., Ohe M.N., Carvalho A.B. (2012). Total parathyroidectomy with presternal intramuscular autotransplantation in renal patients: a prospective study of 66 patients. J Osteoporos.

[27] Echenique-Elizondo M., Díaz-Aguirregoitia F.J., Amondarain J.A., Vidaur F. (2006). Parathyroid graft function after presternal subcutaneous autotransplantation for renal hyperparathyroidism. Arch Surg.

[28] Echenique-Elizondo M., Amondarain J.A., Vidaur F. (2007). Parathyroid subcutaneous pre-sternal transplantation after parathyroidectomy for renal hyperparathyroidism: long-term graft function. World J Surg.

[29] Tanaka Y., Seo H., Tominaga Y., Funahashi H., Matsui N., Takagi H. (1993). Factors related to the recurrent hyperfunction of autografts after total parathyroidectomy in patients with severe secondary hyperparathyroidism. Surg Today.

[30] Neves M.C. das, Rocha L.A. da, Cervantes O., Santos R.O. (2018). Initial surgical results of 500 Parathyroidectomies for Hyperparathyroidism related to chronic kidney disease - mineral and bone disorder. J Bras Nefrol.

[31] Araújo D., Amaral L.M., Guersoni A.C. (2017). Custos do tratamento do hiperparatireoidismo secundário à doença renal crônica com cinacalcete ou paratireoidectomia para pacientes não controlados com a terapia clínica convencional sob a perspectiva do Sistema Único de Saúde. J Bras Econ Saúde.

